# Biological sequences as pictures – a generic two dimensional solution for iterated maps

**DOI:** 10.1186/1471-2105-10-100

**Published:** 2009-03-31

**Authors:** Jonas S Almeida, Susana Vinga

**Affiliations:** 1 Dept Bioinformatics and Computational Biology, University of Texas MD Anderson Cancer Center, Houston, Texas, USA; 2 Instituto de Engenharia de Sistemas e Computadores: Investigação e Desenvolvimento (INESC-ID), R. Alves Redol 9, 1000-029 Lisboa, Portugal; 3 Dept Bioestatística e Informática, Faculdade de Ciências Médicas – Universidade Nova de Lisboa (FCM/UNL), Campo Mártires da Pátria 130, 1169-056 Lisboa, Portugal.

## Abstract

**Background:**

Representing symbolic sequences graphically using iterated maps has enjoyed an enduring popularity since it was first proposed in Jeffrey 1990 as chaos game representation (CGR). The usefulness of this representation goes beyond the convenience of a scale independent representation. It provides a variable memory length representation of transition. This includes the representation of succession with non-integer order, which comes with the promise of generalizing Markovian formalisms. The original proposal targeted genomic sequences only but since then several generalizations have been proposed, many specifically designed to handle protein data.

**Results:**

The challenge of a general solution is that of deriving a bijective transformation of symbolic sequences into bi-dimensional planes. More specifically, it requires the regular fractal nesting of polygons. A first attempt at a general solution was proposed by Fiser 1994 by using non-overlapping circles that contain the polygons. This was used as a starting point to identify a more efficient solution where the encapsulating circles can overlap without the same happening for the sequence maps which are circumscribed to fractal polygon domains.

**Conclusion:**

We identified the optimal inscribed packing solution for iterated maps of any Biological sequence, indeed of any symbolic sequence. The new solution maintains the prized bijective mapping property and includes the Sierpinski triangle and the CGR square as particular solutions of the more encompassing formulation.

## Background

The use of iterative functions to represent nucleotide sequence was originally proposed in 1990 with the designation of Chaos Game Representation, CGR [1]. Since then, the CGR technique evolved from being a graphic representation technique into becoming a platform for pattern recognition [2-9], screening entropic properties [2,10-12], and finally into a generalization of Markov Transition tables [13,14]. That conclusion was further generalized for any symbolic sequence [15] where a dissimilarity metric was also proposed and routes for efficient implementation were established [16]. The emerging interest in alignment-free sequence analysis techniques [17], in particular for application to proteomic sequences, raises the prospect of a wider use of CGR and CGR-related techniques [18,19] capable of exploratory sequence analysis of whole genomes [20-22]. We have subsequently examined the advantage of using non-genomic word-statistics (integer order) for Biological sequence analysis [23] with an application to the SCOP protein database [24]. The strengths of that approach suggest that even more interesting results would be achieved by order-free analysis. However, the potential of hyper-dimensional generalization of CGR such as the use of Universal Sequence Maps, USM [25], are hard to convey and realize in the absence of a 2D projection that retains the bijective mapping property of this technique. This conclusion is particularly clear in a recent report exploring CGR application to aminoacid sequences [26]. In that work, in order to benefit from the scale independency of Chaos Game applied to proteins, the reverse encoding of the aminoacid sequences back to the 4 unit alphabet nucleotide sequences is the solution of choice. Furthermore, as detailed in the derivation of sequence similarity metrics and density kernel functions [13-15], CGR is most useful as a bijective mapping of a sequence of symbols into a numeric vector of the same length, rather than as a technique to compress sequences into individual points. Accordingly, the work reported here is driven by the need to advance the identification of a 2D projection of the vector of CGR positions that is still applicable to longer, often non-genomic, alphabets.

Either as a dimension reduction technique to represent hyper-dimensional CGR [15] or an extension of 2D representation beyond nucleotide sequences, the generalization of bijective iterated maps is desirable for scale-free visualization and analysis of Biological sequences. Three different approaches have been proposed to represent the iterative function results for sequences with more than 4 unique type of units. Those efforts have often been specifically driven towards extending to protein sequences the advantages of CGR of nucleotide sequences.

1. A possible solution is to keep the quadrangular representation for each unit and arrange them in a tabular format [3]. However, this solution sacrifices the regularity of the CGR solution where changing the position of the elements of the alphabet (ACGT) corresponds to a simple translation of the coordinates of the map. In order to preserve equivalence between units of the alphabet, the iterated dividing ratios would have to have the same value for all elements of the larger protein alphabet.

2. To satisfy the desirable equivalence between all elements of the alphabet (all 20 aminoacids in the case of proteins) one could simply keep the 1/2 ratio of the original CGR [1] independently from the length of the alphabet. This solution has in fact been explored [8] by morphing the unit square into a polygon with as many vertices as units in the alphabet. However, this second option sacrifices the bijective mapping property for alphabets with more than 4 units. Without this property, the graphical projection becomes just a visualization technique from which it is no longer possible to completely recover the sequence composition.

3. Finally, the third possibility, chronologically the first extension to be proposed, is to adjust the iterative step such that the prized bijective property is preserved [27], which enables recovery of sequence from map position. However, in that work, the iterative step was adjusted to inscribe the circles where the polygons are inscribed, not the polygons themselves. As a consequence, neither the CGR nor the iconic three alphabet unit map, the Spierpinsky triangle, appear as particular solutions of this formulation.

The study reported here takes the third of these approaches [27] as the starting point and then seeks to identify a new rule that maximizes the packing density of the projection to the point where the polygons are inscribed directly within each other. In addition to providing a more efficient visualization of the sequence, the specific goal of this study if to obtain a solution that also generalizes the 3 alphabet unit solution by the Spierpinsky triangle and the 4 alphabet unit solution of genomic CGR.

## Methods

**Figure 1:**
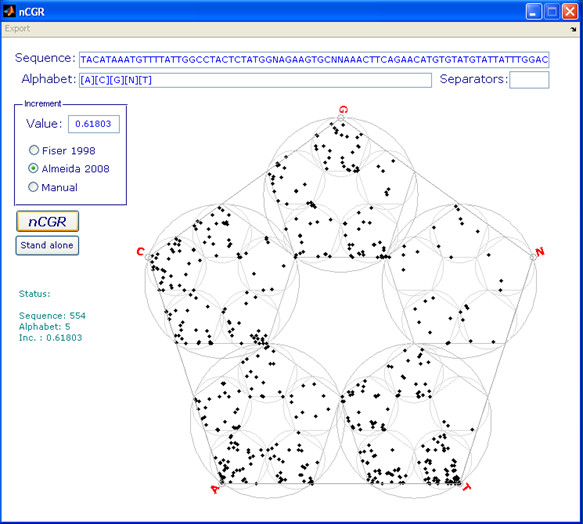
**Snapshot of graphic user interface to Matlab application accompanying this report (see Availability)**. This illustration uses the cDNA sequence Gene Bank index number gi.158122048 obtained through enrichment for up-regulated transcripts in gastric cancer tissue. In addition to the 4 nucleotide identities, this sequence also includes, as per convention, "N" at the positions with undetermined identities.

The algorithms were identified using MathCad (MathSoft Inc) and Matlab (Mathworks Inc) programming environment was used to deploy them. Matlab was also used to produce the graphic displays presented in this report. The pseudo-random number generator of this programming environment was used to produce the 10,000-long random sequences used for the simulations. This manuscript is also accompanied by a graphic user interface (Figure 1) to a Matlab application where arbitrary symbolic sequences can be processed using different dividing ratios, including the generic solution identified by this study.

The main goal of the work described here is achieved with the identification of the dividing ratio in Equation 2 for which maximum packing of nexted, non-ovelapping polygons is obtained. The identification of this equation was achieved empirically with the assistance of MathCad's symbolic processing engine. Specifically, different n-polygons were inscribed in a circle of unitary radius and, using basic trigonometry: the dividing ratio was obtained as the fraction of the distance between any two edges that would identify the perimeter of the target polygon. The observation that the dividing ratio between any two edges is a constant value for each n-polygon was itself an empirical observation. The collection of solutions obtained was symbolically processed using MathCad's equation simplification tool to generate the solution in Equation 2.

## Results

### Current generalization

The starting point for the attempt to find a generic formulation of CGR that is applicable to all alphabets is twofold. Firstly and foremost there is the original CGR formulation for nucleotide sequences [1], using a dividing ratio of 1/2 to fill a unit square domain homogeneously. Secondly, there is the proposition that there is a function of the number of edges in a regular polygon, *n*, that will yield a maximum dividing ratio, s, where the bijective property is still preserved. Fiser et. al [27] proposed Equation 1 as a suitable description of that dependency.

(1) *s *= (1 + sin (*π*/*n*))^-1^

**Figure 2:**
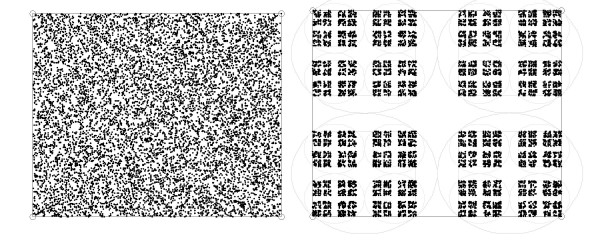
**Comparison of the original, full coverage, Chaos Game Representation (left panel) for nucleotide sequences, which uses a dividing ratio of 1/2, with the sparser implementation suggested by **[27], **described in Equation 1 (right panel), which corresponds to a dividing ratio of 0.586**. The vertices of the square correspond to the 4 nucleotides and the filling was generated by a 10,000 uniformly random synthetic sequence. The circles in the right panel enclose the polygons, to illustrate the property that there is no overlapping at that scale not only for the data points but also between the inclosing circles.

That solution was designed to preserve the bijective mapping property by avoiding overlap between the circles that enclose the individual projections. However, that solution does actually not produce the full packing of the original CGR for n = 4, as noted by the proponents themselves in the original report (color plate 1 in [27]), and graphically recalled in Figure 2. It also sets a looser packing for *n *= 3, where the ratio, *s*, is 0.54, instead of 1/2 as necessary to produce the Sierpinki's triangle.

### Proposed generalization

Here we seek to identify an alternative general formulation for the chaos game dividing ratio parameter, *s*, that will produce the reference structures as special solutions. The relevant reference structures include three maps that use dividing rations of 1/2, for n = 2, 3 and 4. The first is the 1D CGR, i.e. n = 2, used to repeatedly generate hyper-dimensional representations in the Unified Sequence Map technique [15]. The second reference representation is the emblematic Sierpinski triangle [28], for n = 3. Finally and most important, the original formulation of CGR for *n *= 4 [1], where the unit square is covered homogeneously for a dividing ration of 1/2. We have achieved this goal by following a deductive procedure that, while fastidious, was derived using basic trigonometry. The proposed solution was found by noting that a tighter packing would be achieved if instead of inscribing the circles defined by the polygonic projections areas, the inscription was determined for the polygons themselves. The final solution (see Methods for its derivation) is presented in Equation 2:

(2)

## Discussion

**Figure 3:**
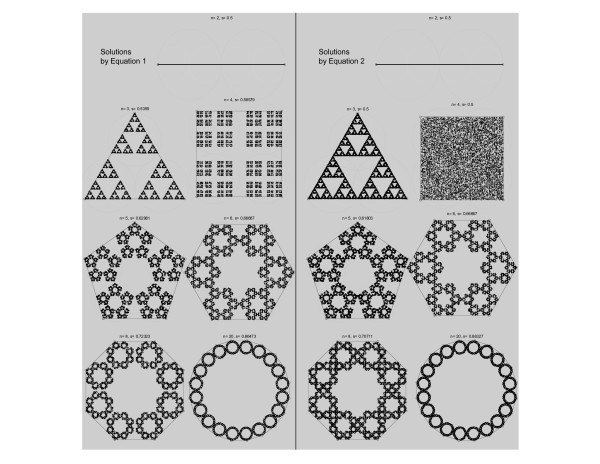
**Graphical comparison of proposed CGR generalization (right, Equation 2) with previously proposed generalization in reference (left, Equation 1)**. The points in each image represent a 10,000-long uniformly distributed random sequence of *n *units. The gray circles involve the corresponding polygons, dark gray for 1^st ^order Markov transition and light gray for 2^nd ^order. In the proposed generalization (right), the polygons do not overlap but the evolving circles may. On the contrary, in the reference generalization in reference (left), the circles do not ever overlap. The desired bijective property of the iterative function is satisfied by both solutions.

The claim that Equation 2 is an accurate generalization of Chaos Game for any alphabet is best first illustrated graphically. In the right hand column of Figure 3, the projection areas defined by the proposed solution are produced for simulated sequences made using alphabets of increasing length. This includes the reference lengths of 2, 3 and 4 where the desired special solutions are correctly defined. For comparison, the formulation that served as starting point for this exercise, Equation 1, is illustrated on the left hand column of the same figure.

It is important to note (see Methods for description of computational derivation) that similarly to the reference circle packing solution by Fiser et. al (Equation 1), the polygon packing achieved by Equation 2 is also an heuristic solution. This is not a rare situation when it comes to fractal geometry [29] where the numerical solutions for a novel graphical configuration are often the starting point, rather than the conclusion, of the deductive process [30]. It nevertheless suggests that further analysis of this solution is needed to uncover simpler and more meaningful patterns.

**Figure 4:**
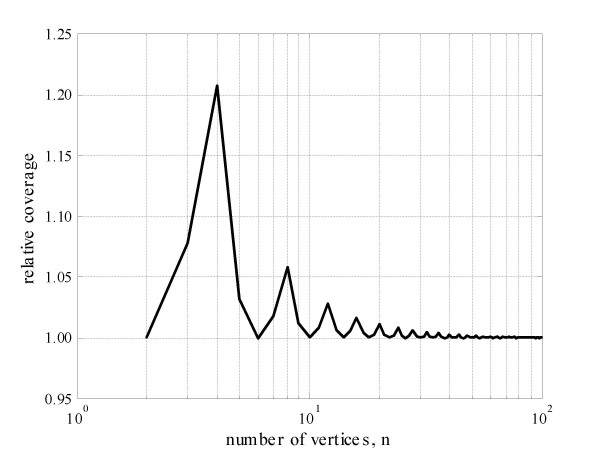
**Improved map coverage by the proposed solution when compared with that of Fiser and co-workers**. The relative coverage is calculated as *(1-s2)/(1-s1)*, where s1 is the solution by Equation 1 and s2 is the proposed solution, by Equation 2. At regular increments of 4 in the length of the alphabet (number of vertices) the two solutions are equivalent, as discussed in the text.

The relative gains of the proposed formulation for different alphabets are assessed in Figure 4. It is noted that the two formulations converge as the alphabet length increases but not monotonically. Instead Figure 4 shows that equivalence between the two formulations occur at regular increments of 4 in the number of vertices of the projection, starting with binary vocabularies (*n *= 2, 6, 10, 14, ...). The explanation for this observation becomes immediately clear when the two solutions are compared graphically, in Figure 3. That figure shows that for the equivalent solutions the vertices shared between distinct polygon areas occur at the exact position where the two involving circles intercept.

As overviewed in the Background section, the enduring popularity of iterated maps to represent Biological sequences has gradually expanded from a convenient graphical illustration into an efficient computational framework for computation. Two routes appear now possible for furthering the CGR transformation. One possibility if to relinquish the requirement to project sequences in two dimensions and instead use unitary hypercube maps [15]. The other possibility assisted by the formulation reported here is to revisit CGR Kernel functions [14] to enable feature detection through entropic profiling [11] for non-genomics sequences. Interestingly, the 4 unit alphabet of genomic code is the exact point where both routes are equivalent, an observation that has been noted, and used, by other authors analyzing non-genomic sequences [26].

## Conclusion

A generic formulation of Chaos Game Representation dividing ratios for 2-dimensional displaying was identified. The new formulation determined ratios for the iterative function that produce the reference representations for alphabets length 2 to 4. Specifically, it produces Sierpinsky's triangle for n = 3 and the original, homogeneously covered Chaos Game representation for n = 4. Given the fact that the density of CGR correspond to a order-free transition matrix – each consecutively nested polygon corresponds to an additional Markov chain order – the value of consistent graphical representation techniques is, potentially, enormous. Furthermore, the growing interest on alignment-free sequence dissimilarity metrics suggests a new role for Chaos Game iterative functions as a scale-free approach to word-statistics. The formulation identified to optimize the dividing ratio is, as is often the case with fractal processes, an empirical result that should be object of further analysis to uncover simpler and more meaningful patterns. Nevertheless, and regardless of the actual CGR computation being performed in two or more dimensions for sequence with longer alphabets, such as proteins, a generic graphical visualization technique is now at hand.

## Availability

The m-code (Matlab, Mathworks Inc) used to generate the figures as well as the application depicted in Figure one are made available with open source at .

## Authors' contributions

JSA identified the optimal packing ration (Equation 2) and wrote the report in close consultation with SV. JSA and SV have jointly performed the exploratory work leading to the three point conceptualization of optimal increment ratios in iterative maps, described at the end of the Background section.
